# Hesitancy among Iranian nursing students regarding future career trajectory: a qualitative analysis

**DOI:** 10.1186/s12960-023-00881-x

**Published:** 2024-01-02

**Authors:** Farnoosh Shafiei, Mostafa Amini-Rarani, Koen Ponnet, Maryam Moeeni

**Affiliations:** 1 School of Management and Medical Information Sciences, Isfahan University of Medical Sciences, Isfahan, Iran; 2 Social Determinants of Health Research Center, Isfahan University of Medical Sciences, Isfahan, Iran; 3 Faculty of Social Sciences, imec-mict-Ghent University, Ghent, Belgium; 4 Health Management and Economics Research Center, Isfahan University of Medical Sciences, Isfahan, Iran

**Keywords:** Preferences, Nursing student, Nursing career, Job choice, Qualitative study

## Abstract

**Background:**

The shortage of skilled nurses is a major concern for health systems worldwide. This may be partly due to the hesitancy of some nurses to enter or remain in the nursing career. This shortage consequently reduces the quality of standard patient care, increases patients’ length of stay in a hospital, increases medical costs, and results in patients’ dissatisfaction. This study aimed to explore hesitancy among senior undergraduate nursing students to pursue a career in nursing.

**Methods:**

This qualitative study adopted a thematic analysis approach. The population comprised senior undergraduate nursing students at Isfahan University of Medical Sciences, Iran, who indicated that they were hesitant to pursue a career in nursing. The study sampling was performed from May 2021 till February 2022 and continued until data saturation. Twenty-four interviews were conducted with the selected students. The attributes related to hesitancy among senior undergraduate nursing students to pursue a career in nursing were extracted as themes and sub-themes.

**Results:**

Four themes were identified: academic idiosyncrasies, individual characteristics, poor nursing market regulations (sub-themes: nursing as a tough and intense career, and unfavorable employment contracts), and the peculiarities of the workplace (sub-themes: conflict within work environment, and barriers to professional nursing practice).

**Conclusions:**

The findings of this study showed that senior undergraduate nursing students weigh their future career options from various academic, personal, professional, and work environment dimensions. The findings provide new insights for decision makers to design and implement innovative strategies to promote retention in nursing careers. We recommend to provide academic counseling for all students and applicants of nursing before they enter the nursing education. Furthermore, we suggest to improve study and work environments, and to implement incentive programs to enhance enthusiasm of nursing students for pursuing a nursing career.

## Background

The World Health Organization designated 2020 as the Year of the Nurse and Midwife due to the paramount role of these careers in promoting global health [1, 2]. It is evident that shortage of nursing staff is a universal challenge with a long history. In 2022, the International Labor Force (ILO) estimated a shortage of about 13 million nurses by 2030 [3, 4]. However, shortage of nurses may be more severe in lower-income countries, especially after the COVID-19 outbreak [4]. A study conducted in 2020 calculated a shortage of 200,000 nurses in Iran [5]. The ratio of nurses per one thousand population in Iran is 1.5, which is much lower than WHO's 4.45 critical staff per 1000 population [6]. Although the shortage of nursing staff has adverse health consequences everywhere (e.g., low quality of standard patient care, a longer hospital stay, higher medical costs, patients dissatisfaction) [7], these consequences are much more severe in lower-middle income countries, as high-income countries can overcome the shortage of nurses by recruiting from poor countries, which is impossible in lower-income countries due to financial incapacity and unfavorable economic situations [5]. Nursing students' hesitancy in pursing the career further is also contributing to this shortage [8].

Existing evidence shows that different countries experience difficulties retaining nurses in their careers [9, 10]. For example, a study conducted on Organization of Economic and Development members showed that although South Korea had the highest number of new graduate nurses per 1000 people in 2019, 16.9% of nursing graduates in this country have changed their jobs from nursing to other fields [11]. Furthermore, a study conducted in South Africa revealed that despite considering incentive policies for nursing students, many students were hesitant to complete their studies, because they were not sure about pursuing a career in nursing. Academic non-performance and financial problems were mentioned as the main reasons [12]. Similarly, the results of a study in Turkey revealed that many nursing students had a negative view of the nursing profession, and were looking for job opportunities other than nursing [13].

Various studies have examined the attributes related to hesitancy among nursing students and graduates with regard to remaining in the nursing career. Research shows that motivational factors, demographic characteristics, sociocultural variables, family preferences, and cognitive factors affect the decision to choose a nursing career from the perspective of nursing students [14–18]. Jeffery proposed a comprehensive model to determine the factors related to the retention of nursing students in their careers. According to the model, various variables, such as emotional factors, academic facilities, academic achievement, psychological aspects, external factors, and professional and workplace characteristics, affect the job decisions of undergraduate and graduate nursing students [19]. In the United States, a large-scale program was developed in 2020 to recruit and retain new nursing students and applicants [20]. The program focuses on overcoming socioeconomic barriers, paying attention to the social determinants of academic achievement, professors’ academic promotion, and expanding nurses’ knowledge, as well as nurses’ social and cultural skills.

Despite the unemployment rate in Iran of approximately 12%, it would be expected that the high chance of getting a job in nursing would be considered an advantage by nursing students; however, this is not the case, and the rate of nurses leaving their jobs is high [21, 22]. Before the COVID-19 pandemic, leaving the job among Iranian nurses was reported as 32.7% in 2018 [23] and 35% in 2013 [24] in two studies. However, the study on nurses from the city of Malayer in Iran found that after the pandemic, 56.41% of the employed nurses intended to leave their jobs [25].

Some studies conducted in Iran have examined the reasons which persuade nurses to remain in the nursing career [26–29]. Most of these studies consider organizational factors, such as salary, level of job satisfaction, workplace conditions, and motivational factors as main reasons to remain in the nursing sector [26–29]. However, to the best of our knowledge, no qualitative study exists on hesitancy to pursue a career in nursing, particularly among nursing students in Iran. Studies conducted outside Iran have indicated that the reasons for hesitancy regarding a future career during nursing education and before beginning a career in nursing can contribute to the retention of nurses in the career [30–32]. Therefore, this study aimed to explore hesitancy among senior undergraduate nursing students affiliated with Isfahan University of Medical Sciences to pursue a career in nursing. The insights from this exploration can provide policy makers and researchers with new evidence on factors influencing decisions of nursing students to remaining in the nursing career.

## Methods

### Setting

This is a qualitative study applying semi-structured interviews. The initial interview guide was designed in the form of open-ended questions. The existing literature was reviewed with the aim of obtaining a comprehensive and adequate understanding of the hesitancy among senior undergraduate nursing students to pursue a career in nursing and to ensure that the interview guide was grounded in existing literature and theory. The initial interview guide was then constructed based on a preliminary set of questions. Thereafter, six interviews were conducted to pilot test the interview guide. Based on the pilot results, some parts of the interview guide were reworded, and several secondary questions were added to the interview guide to promote the transparency and purposefulness of the interview process.

### Data collection

Purposive sampling was performed from May 2021 to February 2022. In total, 67 senior nursing students who enrolled at Isfahan University of Medical Sciences (IUMS), Iran in 2020–2021, of which 24 participated in this study with appropriate diversity in age and gender. All participants stated that they were hesitant to pursue a career in nursing after graduation before starting the interview. Due to the COVID-19 pandemic and the consequent lack of face-to-face access to all participants, we primarily recruited participants from a social group (via WhatsApp messenger) that consisted of undergraduate nursing students. Almost all of undergraduate nursing students at the IUMS used to join the WhatsApp group. We also applied a snowball sampling approach to access the few students who might not have joined the WhatsApp group. We invited the members to participate in the interviews by phone or in person.

In total, 18 of the 24 interviews were conducted over the phone, and 6 interviews were conducted in person at the university campus. No one else was present besides the participant and the interviewer during the interviews.

The interviews were conducted by the first author alone, a postgraduate student who was familiar with the Iranian health sector due to previous experience of working as a health expert in the health centers and has been trained in conducting semi-structured interviews. The interviewer had no prior relationship with the participants. Before starting the interview, the interviewer introduced herself, and briefed the participants about the motivation and objectives of the study. Thereafter, she asked the participants whether or not they were hesitant to pursue a nursing career after graduation. The participants enrolled the current study if they had claimed that they were hesitant to pursue a nursing career after graduation. The interviewee also obtained verbal consent to participate in the interview and to record the interview, and discussed the confidentiality of their names, conversations, etc. This gained the participants’ trust and increased their interest in participating in the study. The sample students neither refused to participate in the study nor dropped out of it. The average time for each interview was about 20 min. All the interviews were audio recorded and then transcribed verbatim by the first author. Transcripts were not returned to sample students for feedback, but the first author checked them against the original recordings to ensure accuracy. Notes were also taken during the interviews. No repeat interviews were carried out. Data collection continued until saturation.

### Analysis

Interviews were analyzed using conventional content analysis [33]. It was conducted as follows: in the first step, three members of the research team independently coded the transcribed data. They read and re-read the transcribed documents and listened to audio-recorded interviews for immersion in data and extracted primary sub–sub-themes, sub-themes, and themes. In the next step, primary themes with initial names were reviewed by the research team. The authors held five meetings to elaborate on conflicts and controversial points and to reach an agreement on emerging themes. The discussion continued until all controversies were addressed. Then, sub–sub-themes, sub-themes, and themes were specified. In the last step, the research team reviewed, modified, and finalized the names of the extracted themes. MAXQDA Analytics Pro 2018 (VERBI GmbH Release 18.2.0 Berlin) was used to manage and analyze the qualitative data.

### Trustworthiness

Criteria suggested by Lincoln and Guba were used to ensure the trustworthiness of the data [34]. During the interviews, important notes were taken and reviewed several times simultaneously with the coding process. To ensure transferability, a series of quotations was embedded in the text, nursing students were selected purposefully, and data were collected and analyzed at the same time. By adopting purposeful sampling, we obtained a various range of nursing students providing diverse perspectives and experiences. This strengthened the transferability by capturing a broader range of nursing students’ insights within the study. Dependability of the study was assured by an auditing approach in which after theme extraction, the authors accompanied by external auditors engaged in complementary comments, cross-checks, investigated inconsistencies, and addressed them to achieve consensus. To improve confirmability, the research team did not overtly allow their values or theoretical inclinations in the conduct of the research or in interpreting the findings derived from it.

## Results

The findings presented herein are the result of a semi-structured interview with 24 nursing students. The interviewees’ genders had an equal distribution: 12 men and 12 women. The minimum age of the interviewees was 21 years, and their maximum age was 37 years. Only one interviewee was married. The interviewees were from 12 Iranian provinces, with 50% from Isfahan Province.

After analyzing the semi-structured interviews, four themes and six sub-themes related to hesitancy among senior undergraduate nursing students to pursue a career in nursing were extracted. The extracted themes included academic idiosyncrasies, individual characteristics, poor nursing market regulations, and the peculiarities of the workplace (Table 1). Each of the extracted themes is explained below.

**Table 1:** Themes and sub-themes extracted from the interviews

Theme	Sub-theme	Sub–sub-theme
Academic idiosyncrasies	Role of professors	Spreading no enthusiasm for Nursing career
		Distant student–professor relationship
Individual characteristics	Personality	Disliking the hospital setting
		Disliking nursing career
Poor nursing market regulations	Nursing as a tough and intense career	Heavy workload
		Lack of sufficient rest and holidays
		High occupational stress
	Unfavorable employment contracts	Low wage
		Job insecurity
		Unprofessional prerequisites for employment
The peculiarities of the workplace	Conflict within work environment	Conflicts among staff nurses
		Conflicts between nurse managers and staff nurses
		Doctor–nurse conflicts
		Patient–nurse conflicts
	Barriers to professional nursing practice	Monotonous tasks
		Lack of autonomy in nursing practice

### Theme 1: academic idiosyncrasies

The first theme involved the characteristics of the academic setting experienced by nursing students. The sub-theme was the role of professors. Some students stated the professors did not use to spread enthusiasm or positive attitude to students and even sometimes talked in negative words about the nursing career. The interviewees called it among the reasons that led them to hesitancy to pursue a career in nursing. One of the interviewees stated:

“*In the first semester, some professors left a very bad impression on us. … In the first session, one of the professors said, ‘You might go to the room of a patient with TB who coughs and transmits it to you*.” (Interviewee 10)

Some students also complained that there was emotionally distant relationship between professors and students. They especially believed the professors did not pay enough attention to the students’ personal and academic problems, nor did they empathize with them or guide them.

“*I told one of the professors that I have to pay the education cost on my own, and sometimes I even cannot afford the bus ticket. But the professor did not understand my problems*.” (Interviewee 22)

### Theme 2: individual characteristics

Some interviewees worried that their individual characteristics may not fit to nursing career. Most of the interviewees considered the incompatibility of the nature of nursing duties with their personalities. They described the hospital setting as painful and full of unfortunate events. For example, one student stated:

“*In this career, you see so many unfortunate events that you become completely callous and ruthless; this is not really compatible with my personality*.” (Interviewee 3)

Some other interviewees reported that they were more interested in other health professions, such as medicine, dentistry, and pharmacy, than nursing. Thus, they were hesitant to pursue a nursing career. One interviewee noted:

“*From the beginning, I was not interested in this major. I didn’t think I would be accepted into this major. That’s why I couldn’t relate to it. I can’t think of any condition or privilege to make me become a nurse*.” (Interviewee 2)

### Theme 3: poor nursing market regulations

The third theme, which was emphasized by most of the interviewees, captures poor nursing market regulations. The nursing students reported that two special dimensions of the nursing career, including toughness and intenseness of nursing, as well as unfavorable employment contracts, discouraged them from pursuing a career in nursing after graduation.

The interviewees also believed that a nursing career is associated with a heavy workload, occupational stress, burnout due to the nature of nursing duties, and not having enough rest and holidays. According to the interviewees:

“*As you work night shifts, you’re not home many nights. And you work when everyone else is on vacation. We’re at work during the Nowruz holiday. Working during holidays is very uncomfortable.* Y*ou have to go to work when everyone else is on vacation.*” (Interviewee 8)

“*This job is very hard. There’s a lot of distress involved. Arguing with patients and their companions and night shifts becomes such a torment that you wouldn’t want to continue the profession*.” (Interviewee 6)

In terms of the risk of burnout, Interviewee 7 remarked:

“*When we start the internship, they have us work as hard as Cosette [a character in the novel Les Misérables]. This makes me feel like I don’t want to stay in the job*.” (Interviewee 7)

Many students point out to unfavorable employment contracts for nursing. some complained about the low wage for nursing careers and believed that nursing wage does not seem to compensate the difficulty and workload of this career.

*“Nurses are not paid fairly; many nurses have financial problems and no longer stay in the profession.”* (Interviewee 19)

Wage discrimination was also mentioned during the interviews. This perceived wage discrimination can play a major role in students’ decisions not to pursue a career in nursing. Two students remarked as follows:

*“Many factors are making me not want to stay in nursing. The most important one is the gap in salaries and not respecting nurses’ rights in Iran.”* (Interviewee 17)

*“One of the senor nurses told me that they have worked so much for this health system, but they were denied even a ticket for recreational accommodations, while other organizations […] have thousands of facilities for their employees.”* (Interview 12)

Many students worried about job insecurity of nursing career. They criticized the employment contracts for nursing careers. According to students’ views, a high percentage of nurses are not working on a fair employment contract. More importantly, many nurses have to work under short-term contracts for many years offering them little or no job security. They believed that it takes a young nurse a very long time and effort to achieve stable employment status. In this context, the interviewees stated:

“*We have no job security. We’re employed on a short-term contract. There could be fired easily, and no one can protest*.” (Interviewee 4)

“*My main problem is income. Salary in nursing is not as high as it is difficult, and I can’t cope with it at all*.” (Interviewee 1)

Another feature of the unfavorable employment contracts for nursing was unprofessional prerequisites for employment, such as marriage status and place of residence. These unprofessional prerequisites discourage students to pursue a nursing career. An interviewee said*:*

“*Another problem with this career of study is that hospitals don’t recruit single nurses anymore. They just employ married nurses*.” (Interviewee 23)

As an additional note, we found some students who compared the nursing market in Iran and that of high-income countries. The interviewees indicated that better living facilities and higher incomes for nurse immigrants could discourage nursing students to work in Iran. Two students said:

“*I’ve heard it is easier to go abroad to study if you’re a nursing student, and that’s why I am studying this major*.” (Interviewee 13)

“*Nursing degrees are much better than other medical degrees for emigration. I’ve heard there’s great demand for nursing in Germany, and they easily recruit nurses from other countries*.” (Interviewee 5)

### Theme 4: the peculiarities of the workplace

The final theme was the environmental conditions related to nursing careers. The conditions that dishearten students from perusing a nursing career after graduation included conflict within nursing work environment, and barriers to professional nursing practice.

Concerning conflict within work environment, the participants believed the conflicts among staff nurses, as well as conflicts of staff nurses with their managers, doctors and patient caused hesitancy among students to peruse a nursing career. Some interviewees worried about competitive attitude of nursing staff, lack of good enough supportive and inclusive environment, and loss of trust among nursing staff.

“*The nursing atmosphere is such that people easily sell each other out. … I mean, if you sit at the station for 10 minutes, you hear people ratting each other out. The atmosphere is not good, and I have no patience for these things*.” (Interviewee 1)

“*I spent my internship at the ICU* [intensive care unit]. *People didn’t get along, and they argued all the time*.” (Interviewee 9)

Some interviewees believed the lack of flexibility and strictness of nurse managers were among main obstacles to their motivation to pursue a nursing career. One of the interviewees noted the following:

“*There’s always a condescending look at our hospital. There’s a head nurse or supervisor who just patrols the ward to find fault with the staff. That’s why many nurses do not renew their contracts*.” (Interviewee 21)

The carelessness of the doctors and underestimating the importance of the nursing career were reasons cited for the reluctance of nursing students to remain in this career. For example, one of the interviewees commented:

*“During the internship, I frequently saw with my own eyes that no attention was paid to the nurses, and the doctors or the hospital managers did not even give a passionate greeting to the nurses.”* (Interview 9)

According to the interviewees’ perspectives, despite the stressful nature and high workload in a nursing career, they are granted less respect and gratitude comparing to the physicians. For example, one of the interviewees stated:

“*There is a huge gap between the job positions of doctors and nurses in Iran. Doctors have the final say in this healthcare system, and this distance is too great. I don’t think it will change any time soon*.” (Interviewee 1)

Furthermore, patient–nurse conflicts were among reasons cited for the reluctance of nursing students to remain in this career. A couple of interviewees noted that nurses regularly experience violence, and lack of empathy and respect from patients and their companions. One interviewee stated:

*“Many people in the hospital think that the nurse is their personal servant. Many of the patient's companions do not show any respect to the nurse and talk to her in a demanding tone.”* (Interviewee 13)

Barriers to professional nursing practice, the second sub-theme related to the workplace, included the monotony of nursing duties, and a lack of autonomy in nursing practice. According to the interviewees, a lack of creative thinking and innovation in performing tasks are examples of the monotony of nursing. One of the interviewees mentioned:

“*Repetitive tasks in nursing can sometimes become annoying. We keep doing the same things over and over again. We keep injecting and placing catheters. I find it repetitive, and I see no progress in it*.” (Interviewee 15)

Some students concerned that nurses have low autonomy and independence in nursing practice. They discussed that a lack of autonomy in nursing practice would decrease job satisfaction among nurses. According to two interviewees:

“*One of the main reasons why I am hesitant to pursue a career in nursing is that you can never put your clinical knowledge into practice*.” (Interviewee 8)

“*There’s really no opportunity in clinical settings to act based on what you’ve learned. Sometimes, you feel like what you’ve learned has no value*.” (Interviewee 14)

## Discussion

In this qualitative study, we explored the reasons behind the hesitance of senior undergraduate nursing students for pursuing a career in nursing. Four themes were extracted: academic idiosyncrasies, individual characteristics, poor nursing market regulations, and the peculiarities of the workplace.

The emotionally distant relationship between professors, and their spreading little enthusiasm and pride on nursing career have made nursing students apathetic toward the profession. In this regard, McLaughlin, Moutray, and Moore (2010) used a qualitative approach to study 68 sophomore undergraduate nursing students in Northern Ireland. Their results showed that the enthusiasm of professors can raise students’ motivation and self-confidence to enter a nursing career [35]. A study of Irish nursing students emphasized the need to create healthcare role models for nursing students to improve the retention of nurses in this career [36]. To promote such role models in Iran, it is necessary to pay attention to elements, such as professors–students relationship. As a role model, professors could strongly contribute to students' motivation for perusing a nursing career.

Another attribute related to the interviewed students’ hesitancy about choosing a career in nursing was personality traits, such as disliking the hospital setting and disliking nursing as a profession. It sees that some students choose the nursing courses unwisely. They may need some guide prior to educational choice to know whether or not their characteristics fit for a nursing career. In some countries, there are programs to familiarize teenagers and young adults with health professions mainly nursing careers. It can help young students make informed choices about their future fields of study. For instance, in the United States, there are programs for high school and college students to attend workplaces and field experience camps, such as “Teens in Nursing” or “Dream Job,” which enable prospective nurses entering nursing programs to have more realistic views [20, 30, 31]. In Iran, however, high school students do not have the opportunity to gain information in their fields of interest before entering university. Neilson and McNally conducted a qualitative study of 20 Scottish high school students who reported no interest in studying nursing after attending field experience programs. They noted that field experience programs need to be undertaken with high quality and in an organized manner [30].

Most of the interviewees in the present study perceived nursing as a tough and intense career. In terms of the toughness of the nursing career, many domestic and foreign studies have reported that occupational stress due to interacting with patients for the long time, and being involved with people’s illness is high among nursing students [37], which has a significant influence on their hesitance about future career [38, 39]. In a similar study by Garside et al. on undergraduate nursing students in the United Kingdom, the results indicated that nursing students had decided not to work as nurses mainly because of irregular and inflexible working hours and, as a result, failing to manage their personal life [40]. Bogossian et al., in a qualitative study on nurses and midwives from Australia, New Zealand, and the United Kingdom, concluded that one of the most important reasons for leaving nursing careers is the working schedule. The authors suggested that if shifts and working hours are wisely organized, nurses can strike an acceptable balance between work and personal life, which can foster their adherence to their career [41]. Garside et al. conducted a study of 114 nurses in the United Kingdom and reported that many interviewees could not afford childcare or did not have the required support networks to take care of their children. The authors suggested that a lack of financial support can discourage nurses from continuing their career [40].

In addition, wage and job security were important attributes making students hesitant to remain in nursing careers in Iran. In two other studies, the students in Turkey [13] and China [42] reported job security and an appropriate job market as prominent motivations for choosing a career in nursing. According to the results of a systematic review, nurses’ salaries are much lower than those of other clinical specialists in Iran [43]. Due to unfavorable employment contracts for nursing career in many low- and middle-income countries, including Iran, the emigration of nurses is prevalent. Existing evidence shows that high-income countries manage to overcome the local shortage of nursing staff by relying on immigrant nurses from low-income countries [11]. However, according to one study on Bahraini nursing students, this pattern of human resource supply has negative consequences for the health system in host countries [43]. To increase the number of nurses, besides increasing the admission of nursing students and facilitating employment conditions, there should be a reasonable balance between the basic salary of nursing practitioners and other members of the healthcare team. Financial incentives could contribute to nurses’ motivation to continue their nursing career.

In this study, workplace conditions played an important role in students’ decision not to choose nursing as their future career. Students often complained about conflicts with colleagues and patients. Liaw et al. examined the factors affecting the choice of a nursing career from the perspective of undergraduate students in Singapore and concluded that independence in decision-making at the workplace is a major factor shaping the career choice of nursing students. The study suggested that the work environment is critical to attracting newly hired nurses [38].

The students in this study also considered the conflicts between patients and nurses as deterrents in continuing their career in nursing. A review study showed that most nurses have experienced at least one type of verbal and physical violence in the workplace [44]. Violence in the workplace has a negative effect not only on the quality of care but also on the job satisfaction of nurses. Consequent job dissatisfaction has been shown to discourage nurses from continuing their career [44]. The other study examined violence in the workplace against nursing students at Arak in Iran and found that about 75% of students had experienced violence the year before the sampling [45]. The findings of one study conducted in Australia, New Zealand, and the United Kingdom reported that nursing students consider the risk of physical or verbal violence in the nursing workplace as a key reason that discourage people from continuing their careers [41].

Nursing students also believed that nursing tasks are often monotonous, and they complained that nurses are not provided enough autonomy and independency. Consistent with these findings, a study on 315 Swedish nursing students showed that creating opportunities for clinical skill development, job diversification, and specialization in clinical affairs were major ways to promote nurses for remaining in their job [46]. Furthermore, in a study conducted in the United Kingdom, nursing students reported that the monotony of nursing tasks and the impossibility of career advancement are reasons that discouraged them from pursuing a nursing career [40].

### Implications for policy

#### The results of this study provided some policy implications:

Academic environment (e.g., professors) could inspire nursing students with great hope and enthusiasm for a nursing career. The nursing schools should promote a friendly academic environment, and a higher emotionally supportive role of professors. Nursing students who study in a pleasant academic environment and feel a strong sense of emotional support from their professors may have more chance to pursue a long-term career in nursing. It is further suggested that responsible organizations such as Ministry of Health and Medical Education, and Ministry of Education develop programs to introduce nursing career, provide academic counseling, and set up scientific/professional camps and visits for all students and applicants of nursing before they enter the nursing education. It can promote their enthusiasm and energy for this career. Nursing students are concerned about the poor nursing market regulations, and the tough and intense duties. Therefore, policymakers either at national or local level need to target these concerns especially through revision of employment regulations in the way to be more supportive and responsible to nurses.

A healthy relationship within work environment can encourage students’ interest in nursing careers. It should be noted that managers or superiors cannot resolve workplace conflicts alone. Instead, all hospital staff, like nursing staff and doctors, should be capable to help one another to resolve or at least mitigate conflicts with peers, superiors, and patients. We suggest to provide nursing students a training program for conflict resolutions. It is also necessary to establish supportive rules to protect all staffs against any kinds of violence.

Further, it is necessary for managers and superiors to provide nurses with an opportunity to have enough autonomy in nursing practice. A system that gives nurses autonomy in nursing practice, and promotes innovation in nursing is inspiring for students.

## Conclusions

The findings of this study showed that senior undergraduate nursing students weigh their future career options from various academic, personal, professional, and work environment dimensions. Considering the specific challenges associated with nursing as a career, they may be hesitant to choose nursing as their long-term career. The findings provide new insights for decision makers to design and implement innovative strategies to promote retention in nursing careers. It is recommended that the Ministry of Health and Medical Education in Iran develops programs to introduce nursing careers, provide academic counseling, and scientific/professional camps and visits for all potential applicants of nursing before they enter the nursing education. It is also necessary to improve the study and work environments, and implement incentive programs to adequately enhance enthusiasm of nursing students for pursuing a nursing career. That may help reduce enormous economic, financial, and psychological costs and their long-term impact on the education system, health system, and society.

## Data Availability

The data sets analyzed during the current study are available from the corresponding author on reasonable request. Raw data are not publicly available to preserve individuals’ privacy.
